# METTL3 promote tumor proliferation of bladder cancer by accelerating pri-miR221/222 maturation in m6A-dependent manner

**DOI:** 10.1186/s12943-019-1036-9

**Published:** 2019-06-22

**Authors:** Jie Han, Jing-zi Wang, Xiao Yang, Hao Yu, Rui Zhou, Hong-Cheng Lu, Wen-Bo Yuan, Jian-chen Lu, Zi-jian Zhou, Qiang Lu, Ji-Fu Wei, Haiwei Yang

**Affiliations:** 1 Department of Urology, The First Affiliated Hospital of Nanjing Medical University, 300 Guangzhou Road, Nanjing, 210029 Jiangsu Province China; 2 Research Division of Clinical Pharmacology, The First Affiliated Hospital of Nanjing Medical University, 300 Guangzhou Road, Nanjing, 210029 Jiangsu Province China

**Keywords:** METTL3, Bladder cancer, m6A, miR221/222, PTEN

## Abstract

**Background:**

METTL3 is known to be involved in all stages in the life cycle of RNA. It affects the tumor formation by the regulation the m6A modification in the mRNAs of critical oncogenes or tumor suppressors. In bladder cancer, METTL3 could promote the bladder cancer progression via AFF4/NF-κB/MYC signaling network by an m6A dependent manner. Recently, METTL3 was also found to affect the m6A modification in non-coding RNAs including miRNAs, lincRNAs and circRNAs. However, whether this mechanism is related to the proliferation of tumors induced by METTL3 is not reported yet.

**Methods:**

Quantitative real-time PCR, western blot and immunohistochemistry were used to detect the expression of METTL3 in bladder cancer. The survival analysis was adopted to explore the association between METTL3 expression and the prognosis of bladder cancer. Bladder cancer cells were stably transfected with lentivirus and cell proliferation and cell cycle, as well as tumorigenesis in nude mice were performed to assess the effect of METTL3 in bladder cancer. RNA immunoprecipitation (RIP), co-immunoprecipitations and RNA m6A dot blot assays were conducted to confirm that METTL3 interacted with the microprocessor protein DGCR8 and modulated the pri-miR221/222 process in an m6A-dependent manner. Luciferase reporter assay was employed to identify the direct binding sites of miR221/222 with PTEN. Colony formation assay and CCK8 assays were conducted to confirm the function of miR-221/222 in METTL3-induced cell growth in bladder cancer.

**Results:**

We confirmed the oncogenic role of METTL3 in bladder cancer by accelerating the maturation of pri-miR221/222, resulting in the reduction of PTEN, which ultimately leads to the proliferation of bladder cancer. Moreover, we found that METTL3 was significantly increased in bladder cancer and correlated with poor prognosis of bladder cancer patients.

**Conclusions:**

Our findings suggested that METTL3 may have an oncogenic role in bladder cancer through interacting with the microprocessor protein DGCR8 and positively modulating the pri-miR221/222 process in an m6A-dependent manner. To our knowledge, this is the first comprehensive study that METTL3 affected the tumor formation by the regulation the m6A modification in non-coding RNAs, which might provide fresh insights into bladder cancer therapy.

**Electronic supplementary material:**

The online version of this article (10.1186/s12943-019-1036-9) contains supplementary material, which is available to authorized users.

## Introduction

Bladder cancer (BCA) has become the fifth most common cancer in USA, with 81,190 estimated new cases in 2018 [1]. The development of bladder cancer is a complex process, which is caused by abnormal genetic changes and/or epigenetic abnormalities. Epigenetic abnormalities mainly occur in many levels including DNA [2], RNA [3] and histone modification [4–7]. In RNA levels, more than 100 types of post-transcriptional modifications have been identified. Among them, N6-methyladenosine (m6A) RNA methylation is one of the most common modifications, accounting for about 50% of total methylated ribonucleotides and 0.1–0.4% of all adenosines in total cellular RNAs [8, 9]. In bladder cancer, Cheng.et al has verified that the m6A level in tumor tissues was significantly elevated. They further found that methyltransferase-like 3 (METTL3), an main component in the so-called m6A ‘writer’, could promote bladder cancer progression via AFF4/NF-κB/MYC signaling network by an m6A dependent manner [10].

METTL3 is known to be involved in all stages in the life cycle of RNA. It plays a pivotal role in pre-mRNA splicing [11, 12], 3′-end processing [13, 14], nuclear export [15–17], translation regulation [18, 19], messenger RNA (mRNA) decay [20–22], and microRNA (miRNA) processing [23, 24]. Accordingly, METTL3 affects the tumor formation by the regulation the m6A modification in the mRNAs of critical oncogenes or tumor suppressors [25–28]. At the level of non-coding RNAs, METLL3 could promote the formation of m6A-circRNAs, exhibiting distinct patterns of m6A modifications compared to mRNAs [29] . Moreover, it could affect the internal m6A modification of lincRNA1281 and mediate a competing endogenous RNA (ceRNA) model to regulate mouse embryonic stem cell differentiation [30].Alarcon et al found that METTL3 could promote the maturation of miRNAs (let-7e, miR221/222, miR4485, miR25, miR93, miR126 and miR335 et al) by interact with the microprocessor protein DGCR8 [24]. These suggested that METLL3 could exhibit its roles in normal human physiological process or diseases by regulating the m6A modification of the non-coding RNAs. However, whether this mechanism is related to the proliferation of tumors induced by METTL3 is not reported yet.

In the present study, we indeed found METTL3 exhibited its oncogenic role in bladder cancer through interacting with the microprocessor protein DGCR8 and positively modulating the pri-miR221/222 process in an m6A-dependent manner. Moreover, high expression of METTL3 was related to poor prognosis in bladder cancer patients. It implies that METTL3 may serve as a novel prognostic and/or therapeutically target in bladder cancer.

## Materials and methods

### Clinical specimens

Bladder cancer tissues and their paired normal tissues were obtained from patients who were diagnosed with bladder cancer and undergone surgery in the First Affiliated Hospital with Nanjing Medical University (Jiangsu Province Hospital) between 2010 and 2013. The follow-up deadline was January 2018. All patients signed informed consent before using clinical materials. The use of tissues for this study has been proved by the ethics committee of the First Affiliated Hospital with Nanjing Medical University (Jiangsu Province Hospital).

### Cell culture

The bladder cancer cell lines EJ and T24 were obtained from the Type Culture Collection of the Chinese Academy of Sciences (Shanghai, China). All cells were cultured in DMEM medium containing 10% fetal bovine serum (FBS; Gibco, Australia) and 1% penicillin–streptomycin with 5% CO_2_ at 37 °C in a humidified incubator.

### Transfection

The lentivirus constructing of METTL3 knockdown or overexpress was obtained from OBIO (Obio Technology Corp, China). Bladder cancer cells were plated in 6 wells dishes at 50% confluence and infected with METTL3 overexpression lentivirus (termed as oeMETTL3), a negative control (termed as NC), METTL3 knockdown lentivirus (termed as shMETTL3–1, shMETTL3–2), or a scramble control (termed as shNC) in T24 and EJ cells, respectively. Pools of stable transductions were generated by selection using puromycin (4 μg/ml) for 2 weeks.

All miRNA mimics used for transfection were purchased from GenePharma (Shanghai, China). Transfections were performed using the Lipofectamine 2000 kit (Invitrogen, USA) according to the manufacturer’s instructions.

### Tissue microarray (TMA) and immunohistochemistry (IHC)

TMA (16 × 10) was constructed from 180 cases of formalin-fixed, paraffin-embedded bladder cancer tissues. IHC was performed on TMA to evaluate METTL3 protein expression. TMAs were treated with xylene and 100% ethanol, followed by decreased concentrations of ethanol. After antigen retrieval, TMAs were blocked and stained with anti-METTL3 antibody (1:200, Abcam, USA), followed by secondary antibody incubation and standard avidin biotinylated peroxidase complex method. Hematoxylin was used for counterstaining, and images were obtained with an upright microscope system (Nikon, JAPAN). The total METTL3 immunostaining score was calculated as the sum of the score for the proportion of positively stained tumor cells (PP) and the score for staining intensity (SI) given by two pathologists blinded to the clinical parameters. PP was scored into four categories: 0 (< 5%, negative), 1 (5–25%, sporadic), 2 (25–50%, focal), 3 (> 51%, diffuse) and SI was scored on a scale of 0 to 3 (0, negative staining; 1, weak staining; 2, moderate staining; 3, strong staining). The final staining score was calculated by multiplying SI and PP score, resulting in a score value ranging from 0 to 9. The positive level of IHC staining was scored by two urologists and patients with different scores were divided into low- (0–3) and high-staining (4–9) groups. All information of patients was supplied in Additional file 1: Table S1

### RNA extraction and quantitative real-time PCR (qRT-PCR)

Total RNA was isolated from tissues and cells by using Trizol reagent (Invitrogen, USA) according to the manufacturer’s protocol. cDNA was synthesized using HiScript II (Vazyme, China) and qRT-PCR for mRNA and miRNA was performed on StepOne Plus Real-Time PCR system (Applied Biosystems, USA) or LightCycler 480 (Roche, USA). U6 and β-actin were used as an internal standard control for miRNA and mRNA detection, respectively. Each sample was replicated three times and data was analyzed by comparing Ct values. All PCR primers were purchased from GeneCopoeia (Guangzhou, China)and listed in Additional file 2: Table S2.

### Western blot

Total cellular proteins were lysed by RIPA buffer containing protease inhibitors (Sigma, USA). The protein extractions were harvested and quantified by bicinchoninic acid (BCA) analysis (Beyotime, China). Protein extractions were separated by 10% SDS-PAGE and transferred onto polyvinylidene fluoride (PVDF) membranes (Millipore, USA). After the incubation with a high affinity anti-METTL3 antibody (1:1000, Abcam, USA), anti-DGCR8 antibody (1:1000, Abcam, USA), anti-PTEN antibody (1:1000, Abcam, USA), anti-β-actin antibody (1:1000, Cell Signaling Technology, USA) or anti-GAPDH antibody (1:1000, Cell Signaling Technology, USA), the membranes were then incubated with peroxidase (HRP)-conjugated secondary antibody (1:1000, Cell Signaling Technology, USA). After washes, signals were detected using a chemiluminescence system (Bio-Rad, USA) and analyzed using Image Lab Software.

### Cell proliferation and colony formation assay

For cell proliferation assay, the transfected cells were seeded into 96-well plates at a density of 2000 cells per well. At 0, 24, 48, 72 and 96 h after seeding, cell viability was measured by the cell counting kit-8 (CCK-8) system (Dojindo, Japan) according to the manufacturer’s instructions. Briefly, each well was added with 10 μl CCK-8 solution and the plate was incubated at 37 °C for 1 h in dark. The absorbance was measured at 450 nm with a microplate reader (Tecan, Switzerland).

For colony formation assay, the transfected cells were seeded into 6-well plates at a density of 700 cells per well and maintained in DMEM medium containing 10% FBS for 2 weeks. After fixed with methanol, the cells were stained with 0.1% crystal violet 30 min and then the colonies were imaged and counted.

### Cell cycle assay

1 × 10^6^ cells were collected, washed with phosphate buffer saline (PBS) and fixed with 75% cold ethanol for 24 h at − 20 °C. After washed with PBS twice and stained with propidium iodide by the cycletest plus DNA reagent kit (BD Biosciences, USA) at room temperature for 30 min, cells were assessed by flow cytometry (Becton Dickinson, USA) and cell cycle would be analyzed by Cell Quest Modfit software.

### RNA m6A dot blot assays

The Poly(A) + RNAs were firstly denatured by heating at 65 °C for 5 min and transferred onto a nitrocellulose membrane (Amersham, GE Healthcare, USA) with a Bio-Dot apparatus (Bio-Rad, USA). The membranes were then UV cross-linked, blocked, incubated with m6A antibody (1:1000, Abcam, USA) overnight at 4 °C and subsequently incubated with HRP-conjugated goat anti-mouse IgG (1:3000, Proteintech, USA). Finally, the membrane can be visualized by the chemiluminescence system (Bio-Rad, USA). The membrane stained with 0.02% methylene blue (MB) in 0.3 M sodium acetate (pH 5.2), was used to ensure consistency among different groups.

### Dual-luciferase reporter assay

Cells were co-transfected with plasmids containing 3′-UTR of wild or mutant fragments from PTEN (gene of phosphate and tension homology deleted on chromsome ten) and miRNA mimics using Lipofectamine 3000 (Invitrogen, Foster city, CA) according to the manufacturer’s protocol. At 24 h after transfection, firefly and renilla luciferase activities were measured consecutively by using dualluciferase reporter assay system (Promega, Massachusetts, USA). Finally, ratios of luminescence from firefly to renilla luciferase were calculated.

### m6A RNA immunoprecipitation assay (MeRIP)

T24 cells stably transfected with either the METTL3 overexpress lentiviral or control were UV-irradiated at 254 nm, 400 mJ/cm^2^ (Stratagene Stratalinker), and lysed with RIP lysis buffer (Magna RIP Kit, Millipore, MA) at 4 °C via disruptive sonication. Immunoprecipitations of endogenous DGCR8 were performed using an anti-DGCR8 antibody (1:1000, Abcam, USA) overnight at 4 °C. After washing, the immunoprecipitated protein-RNA complex was analyzed by western blot and treated with Proteinase K. RNAs were extracted by phenol: chloroform: isoamyl alcohol and subjected to qRT-PCR using primers for pri-miRNAs and normalizing to input.

For the m6A RNA binding experiments, the RNAs of T24 cells stably transfected with either the METTL3 overexpress lentiviral or control were isolated and treated with DNase I (Sigma Aldrich, USA). RNAs were fragmented by sonication for 10 s on an ice water mixture. Immunoprecipitations were performed using an anti-m6A antibody (1:1000, Abcam, USA) previously bound to magnetic Dynabeads (Life Technologies, USA) in the RIP Immunoprecipitation buffer (Magna RIP Kit, Millipore, MA) and incubated with DNA-free fragmented RNAs. Beads were then treated with Proteinase K (20 mg/ml) for 1.5 h at 42 °C. RNAs was extracted by phenol: chloroform: isoamyl alcohol and subjected to qRT-PCR using primers for pri-miRNAs and normalizing to input.

### Co-immunoprecipitations assay

Co-immunoprecipitation was performed in T24 cells using PierceTM Co-Immunoprecipitation Kit (ThermoFisher Scientific, USA) according to the manufacturer’s protocol. Briefly, immunoprecipitations of METTL3 were performed using an anti-METTL3 antibody (1:1000. Abcam, USA) overnight at 4 °C. After washing, the immunoprecipitated complex was treated with either RNase A or RNase inhibitor (Sigma Aldrich, USA) 5 min at 37 °C. Then anti-DGCR8 (1:1000, Abcam, USA) was used for western blot analysis.

### Xenografts in mice

The T24 cells were stably transfected with shMETTL3, oeMETTL3 or their negative control. About 1× 10^7^ cells were injected subcutaneously into the axilla of the female athymic BALB/C nude mice (4–6 weeks old, 18–22 g, five mice per group). Tumor growth was monitored every week by measuring the width (W) and length (L) with calipers, and the volume (V) of the tumor was calculated using the formula V = (W^2^ × L)/2. Four weeks after injection, the mice were euthanized and tumors were removed, weighed, fixed, and embedded for IHC. The animal studies were performed in accordance with the institutional ethics guidelines for animal experiments, which was approved by the animal management committee of Nanjing Medical University.

### Statistical analysis

Data were analyzed using SPSS version 19.0 and presented as means ± standard deviation (SD). Student’s t-test was performed to analyze differences between the two groups, and *P* < 0.05 was considered to indicate statistical significance. All experiments were repeated more than three times, and each experiment was performed in triplicate.

## Results

### METTL3 was upregulated in bladder cancer and correlated with prognosis of bladder cancer patients

**Fig. 1:**
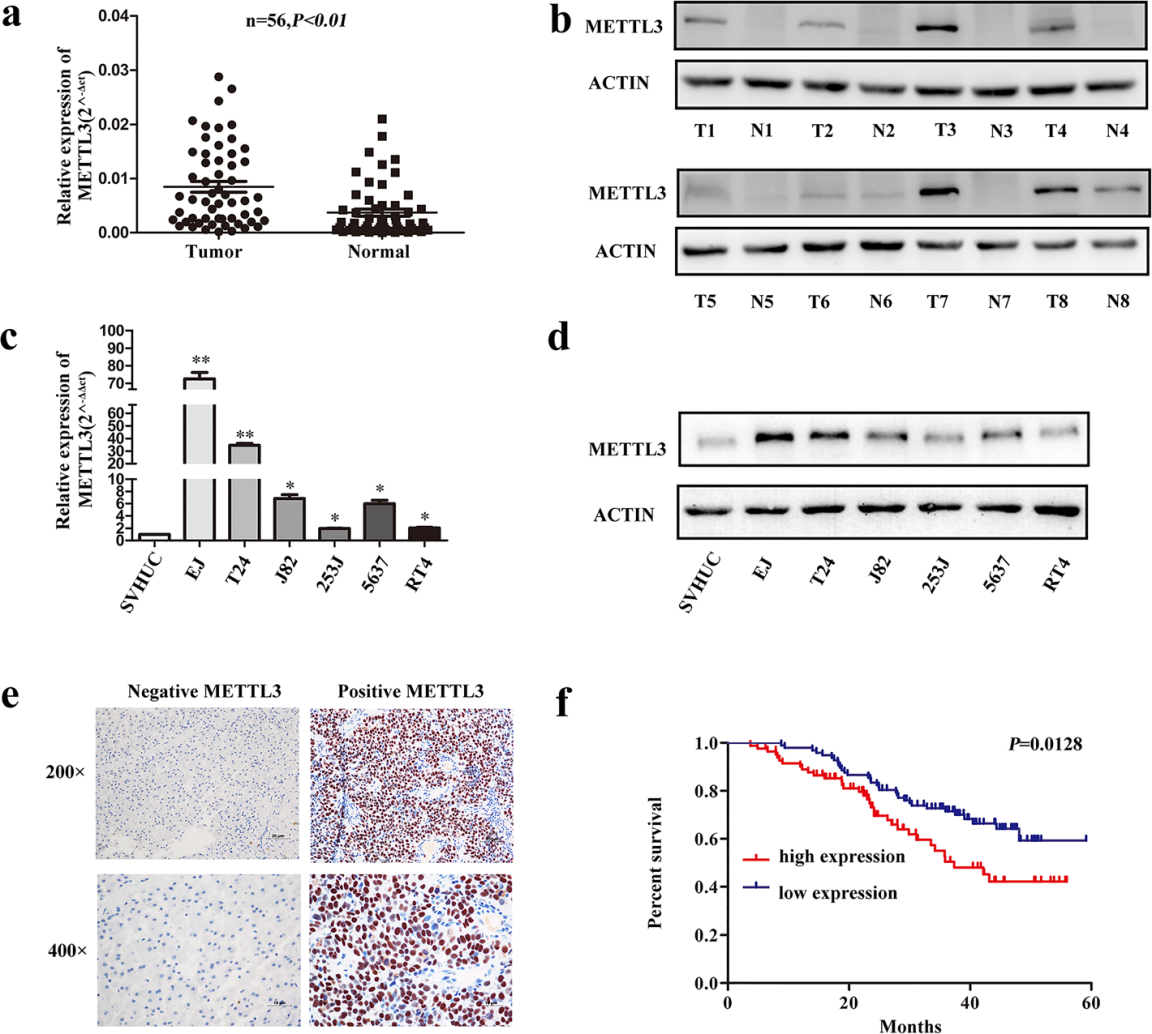
METTL3 was up-regulated in bladder cancer tissues and cell lines and served as a prognostic factor for bladder cancer patients. **a**. Relative expression of METTL3 mRNA in 56 paired fresh bladder cancer tissues (Tumor) and matched adjacent normal tissues (Normal) quantified by qRT-PCR. METTL3 was expressed significantly higher in bladder cancer tissues compared with that in adjacent normal tissues (*P* < 0.01). **b**. The expression of METTL3 protein in 8 paired bladder cancer tissues (T) and adjacent normal tissues (N) by western blot. **c** and **d**. Relative expression level of METTL3 in bladder cancer cell lines and SV-HUC cell was used as control used by qRT-PCR and western blot. **e**. IHC analysis of METTL3 in bladder cancer tissue microarray at 200× and 400× magnification. Scale bars indicated 20 μm and 10 μm. **f**. Kaplan-Meier survival curves of overall survival in 180 bladder cancer patients based on METTL3 expression analyzed by IHC staining. The log-rank test was used to compare differences between two groups (*P* = 0.0128)

METTL3 was significantly upregulated in bladder cancer tissues, compared with the adjacent tissues (Fig. 1a), which was consistent with the results from tumor samples with detailed clinical information which were downloaded from TCGA database (https://cancergenome.nih.gov) (Additional file 3: Figure S1a). The protein level of METTL3 in bladder cancer tissues also increased significantly, compared with that in the adjacent normal tissues (Fig. 1b). We found METTL3 was upregulated in bladder cancer cell lines, compared with that in the normal urinary epithelial cell line SV-HUC, both in mRNA and protein levels(Fig. 1c,d). IHC analysis in bladder cancer tissues showed that the expression of METTL3 was related to tumor histological grade (Table 1). The histological grade was higher in the high expression of METTL3 group. Furthermore, Kaplan-Meier analysis showed that bladder cancer patients with high expression of METTL3 had worse prognosis and shorter survival time, compared with those with low expression of METTL3 (Fig. 1f). However, at the level of its mRNA, the prognostic analysis of METTL3 from tumor samples with detailed clinical information which were downloaded from TCGA database showed a trend of adverse prognosis in both METTL3 and bladder cancer, but it had no statistical significance (Additional file 3: Figure S1b).

**Table 1:** Correlation between METTL3 expression and clinicopathological characteristics of 180 BCa patients

Parameters	Number of cases	METTL3 expression		*P* Value
		Low	High	
All cases	180	97	83	
Age(years)				
<65	76	43	33	0.536
≥65	104	54	50	
Gender				
Male	141	73	68	0.279
Female	39	24	15	
TNM stage				
pTa-pT1	116	64	52	0.642
pT2-pT4	64	33	31	
Histological grade				
Low	78	50	28	0.016^a^
High	102	47	55	
Tumor size(cm)				
<3	114	66	48	0.156
≥3	66	31	35	

^a^Statistically significant, *P*<0.05

Notes: The total METTL3 immunostaining score was calculated as the sum of the score for the proportion of positively stained tumor cells (PP) and the score for staining intensity (SI) given by two pathologists blinded to the clinical parameters. PP was scored into four categories: 0 (< 5%, negative), 1 (5–25%, sporadic), 2 (25–50%, focal), 3 (> 51%, diffuse) and SI was scored on a scale of 0 to 3 (0, negative staining; 1, weak staining; 2, moderate staining; 3, strong staining). The final staining score was calculated by multiplying SI and PP score, resulting in a score value ranging from 0 to 9. The positive level of IHC staining was scored by two urologists and patients with different scores were divided into low- (0–3) and high-staining (4–9) groups

### Knockdown of METTL3 inhibited the proliferation of bladder cancer in vivo and in vitro

Two bladder cancer cell lines, T24 and EJ, were stably transfected with knockdown lentivirus, and control lentivirus. The expression of METTL3 was then confirmed with qRT-PCR and western blot (Additional file 4: Figure S2a, b, c and d).

**Fig. 2:**
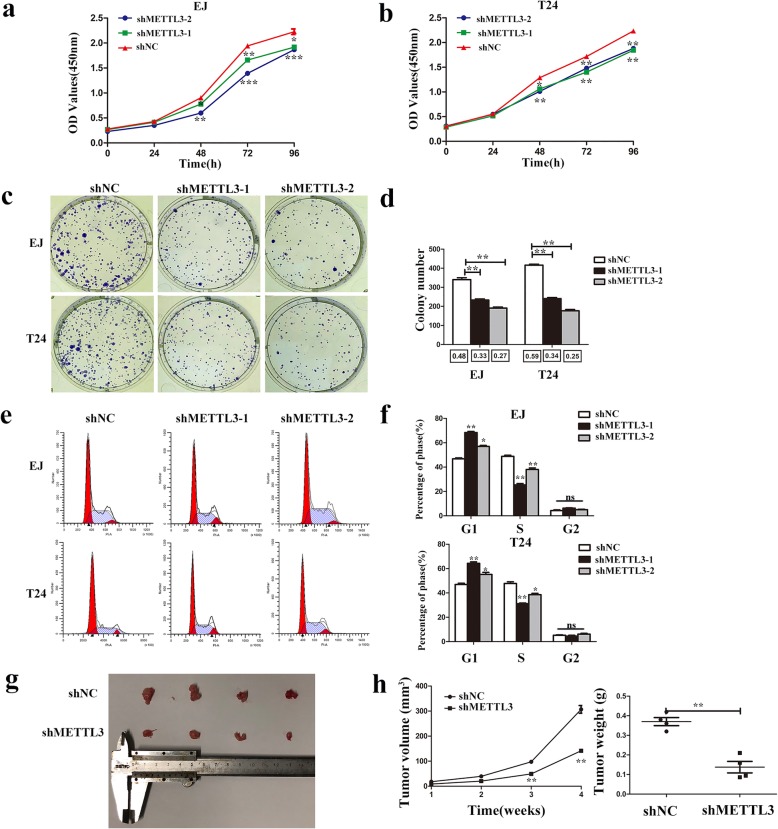
Knockdown of METTL3 inhibited bladder cancer proliferation in vitro and in vivo***.*** **a** and **b**. Knockdown of METTL3 inhibited cell proliferation as indicated by CCK-8 assays in EJ and T24 cells. **c** and **d**. Colony formation assay showed that METTL3 knockdown significantly decreased the cloning number of T24 and EJ cells compared with control group. The colony formation rate (colony number/ cells per well) is shown below the histogram. **e** and **f**. Cell cycle analyzed by flow cytometry. Histogram showed that METTL3 knockdown arrested at G1 phase in T24 and EJ cells. **g**. Representative image of the nude mice injected with T24 cells. h. Tumor weight and the tumor growth curve were measured in METTL3 knockdown cell and its control group. Data represented the mean ± SD from three independent experiments, *P < 0.05, **P < 0.01

The CCK8 assays showed that METTL3 knockdown led to significantly decreased cell proliferation (Fig. 2a, b). The colony formation assays also displayed that knockdown of METTL3 decreased the colony formation efficiency (Fig. 2c, d). Furthermore, the flow cytometry analysis showed that the percentage of G1 increased and the percentage of S phase decreased in METTL3 knockdown cells (Fig. 2e, f). In xenograft models, the tumors injected with knockdown of METTL3 (shMETTL3)cells grew more slowly than those injected with control cells (shNC) (Fig. 2g,h).

### Overexpression of METTL3 promoted proliferation of bladder cancer in vivo and in vitro

**Fig. 3:**
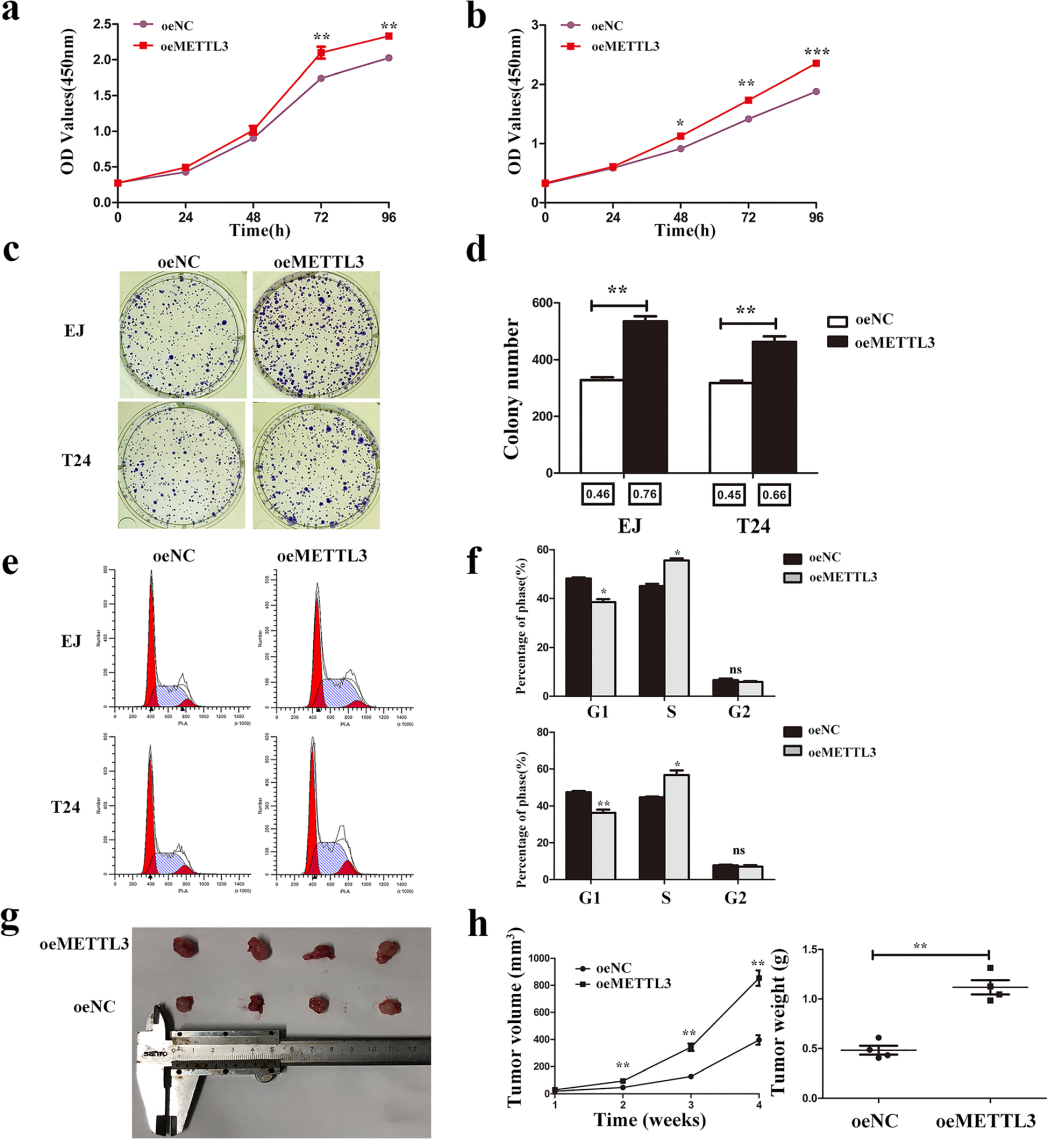
Overexpression of METTL3 promoted bladder cancer proliferation in vitro and in vivo*.* **a** and **b**. Overexpression of METTL3 promoted cell proliferation as indicated by CCK-8 assays in EJ and T24 cells. **c** and **d**. Colony formation assay showed that METTL3 overexpression significantly increased the cloning number of T24 and EJ cells compared with control group. The colony formation rate (colony number/ cells per well) is shown below the histogram. **e** and **f**. Cell cycle analyzed by flow cytometry. Histogram showed that METTL3 overexpression decreased the percentage of G1 phase and increased the percentage of S phase. Data represented the mean ± SD from three independent experiments, *P < 0.05, **P < 0.01. **g**. Representative image of the nude mice injected with T24 cells. **h**. Tumor weight and the tumor growth curve were measured in METTL3 overexpression cells and its control group. Data represented the mean ± SD from three independent experiments, *P < 0.05, **P < 0.01

The CCK8 assays indicated that overexpression of METTL3 significantly promoted cell growth compared with the oeNC group (Fig. 3a, b). The colony formation assays also displayed that colony formation efficiency was increased in METTL3 overexpression cells (Fig. 3c, d). Furthermore, the flow cytometry analysis showed that the percentage of G1 phase decreased and the percentage of S phase increased in METTL3 overexpression cells (Fig. 3e, f). Moreover, the tumors injected with METTL3 overexpression (oeMETTL3) cells grew more rapidly than those injected with control cells (oeNC) in subcutaneous xenograft tumor model (Fig. 3g, h).

### METTL3 regulated the processing of miR221/222 by DGCR8

**Fig. 4:**
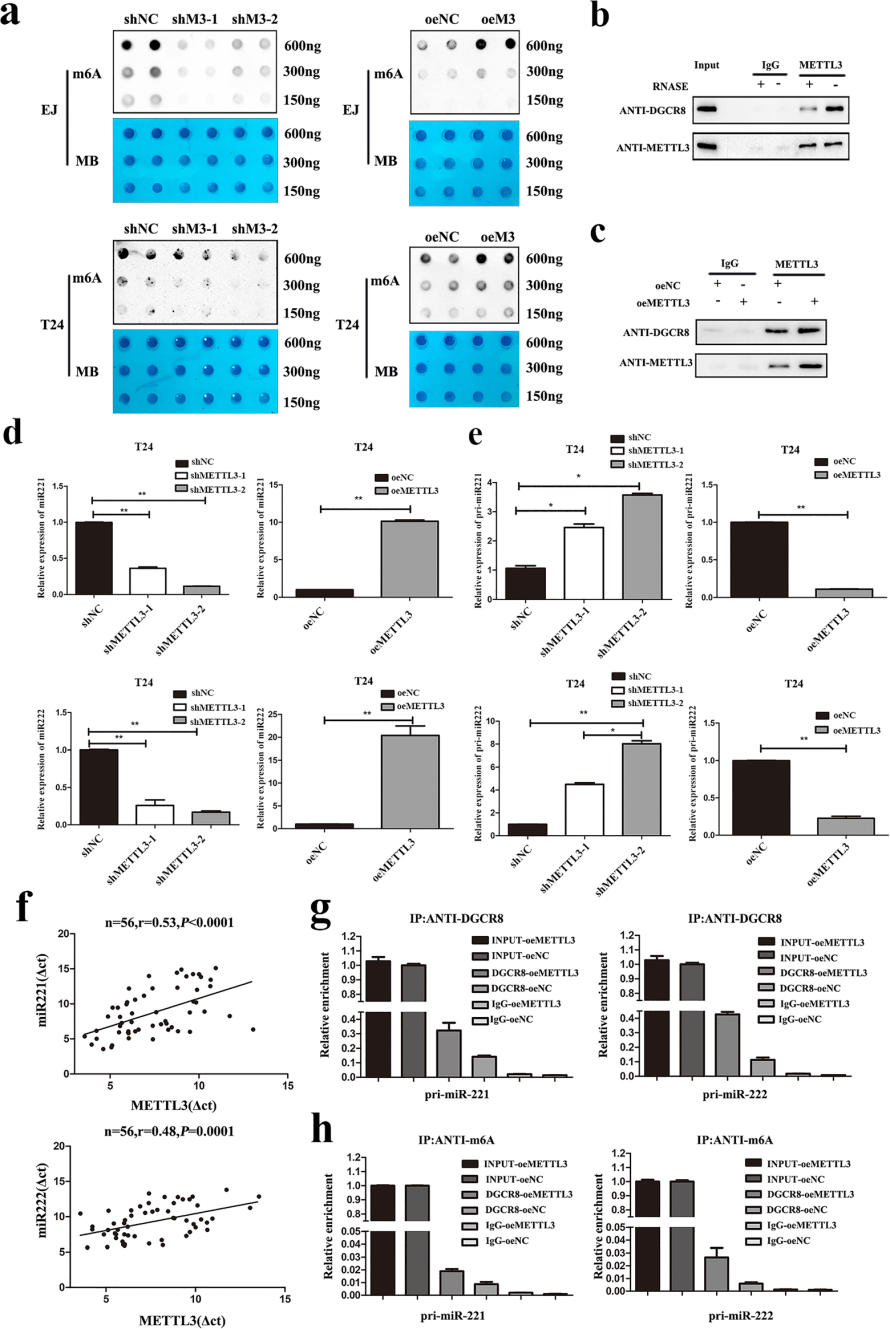
METTL3 interacted with the microprocessor protein DGCR8 and modulated the pri-miR221/222 process in an m6A-dependent manner. **a**. m6A dot blot assays of EJ and T24 cells with knockdown or overexpression of METTL3. Methylene blue (MB) stain as loading control. It was obvious that the methylation of RNA decreased significantly after METTL3 knockdown, while increased significantly after METTL3 overexpression. **b**. Co-immunoprecipitation of the METTL3-interacting protein DGCR8. Cells were UV-cross-linked before the immunoprecipitation. Western blot using the DGCR8 and METTL3 antibodies and IgG used as control for the IP. **c**. Immunoprecipitation of DGCR8, METTL3 and associated RNAs from control cells or METTL3 overexpression cells. Cells were UV-cross-linked before the immunoprecipitation. Western blot or Immunoblot were conducted using the antibodies described above. **d**. The expression of miR221/222 were verified by qRT-PCR in METTL3 knockdown and overexpression cells. **e**. The expression of pri-miR221/222 were verified by qRT-PCR in METTL3 knockdown and overexpression cells. **f**. A moderate positive correlation between the expression of METTL3 and miR221/222 was showed in bladder cancer tissues by qRT-PCR. **g**. Detection of pri-miRNA binding to DGCR8 by immunoprecipitation of DGCR8-associated RNA from control and METTL3 overexpression cells followed by qRT-PCR. **h**. The detection of pri-miRNAs m6A modification level by immunoprecipitation of m6A modified miRNA in control or METTL3 overexpression cells followed by qRT-PCR. Data represented the mean ± SD from three independent experiments, *P < 0.05, **P < 0.01

The m6A dot blot showed that METTL3 knockdown significantly decreased the m6A level. On the contrary, the m6A level was increased by METTL3 overexpression in both T24 and EJ cell lines (Fig. 4a). Previously, Alarcon R.et al found METTL3 could interact with the microprocessor protein DGCR8 and positively modulate the pri-miRNA process. So, we assessed whether METTL3 was required for the engagement of pri-miRNAs by the DGCR8 in bladder cancer. Immunoprecipitation assay showed that METTL3 could bind to DGCR8. Moreover, RNase treatment weakened the binding of METTL3 and DGCR8, suggesting that their binding might be partly mediated by miRNAs (Fig. 4b). We also observed a significant increase in miRNAs bound by DGCR8 in METTL3 overexpression cells (Fig. 4c), confirming that METTL3 could affect the binding of DGCR8 to m6A methylated miRNAs. All these results implied that METTL3 could affect pri-miRNAs processing by regulating the recognition and binding of DGCR8 to pri-miRNAs in bladder cancer. Former study found the main miRNAs modulated by METTL3 significantly included miR221/222, let-7e, miR25, miR93, miR126, miR335, miR4485 and et al [24]. We found that only miR221/222, miR225, miR4485 and let-7e were altered in T24 after METTL3 knockdown (Additional file 5: Figure S3a). Then, we predicted the prognosis of these microRNAs in bladder cancer in the database (http://kmplot.com/analysis/) and found that only miR221/222 played an oncogenic role in bladder cancer (Additional file 5: Figure S3b).

Then, we tested whether METTL3 exhibited its oncogenic role by regulating the expression of miRNA221/222 in bladder cancer. Firstly, we found miR221/222 decreased significantly in METTL3 knockdown cells and upregulated in METTL3 overexpression cells (Fig. 4d). Accumulation of pri-miR221/222 occurs in METTL3 knockdown cells and decreased in METTL3 overexpression cells (Fig. 4e). The correlation analysis revealed a positive correlation between the expression of METTL3 and miR221 (r = 0.2928, *P* < 0.01) or miR222 (r = 0.2386, *P* < 0.01) in 56 tumor tissues (Fig. 4f). Moreover, we found an increased level of pri-miR221/222 binding by DGCR8 immunoprecipitated from METTL3 overexpression cells (Fig. 4g). Moreover, MeRIP revealed that METTL3 overexpression significantly increased the amount of pri-miR221/222 modified by m6A (Fig. 4h). Taken together, these results indicated that the METTL3 could enhance the recognition of pri-miR221/222 by DGCR8 and the subsequent processing to mature miRNAs in an m6A manner.

### miR221/222 interference rescued the proliferation induced by METTL3 in bladder cancer cells

**Fig. 5:**
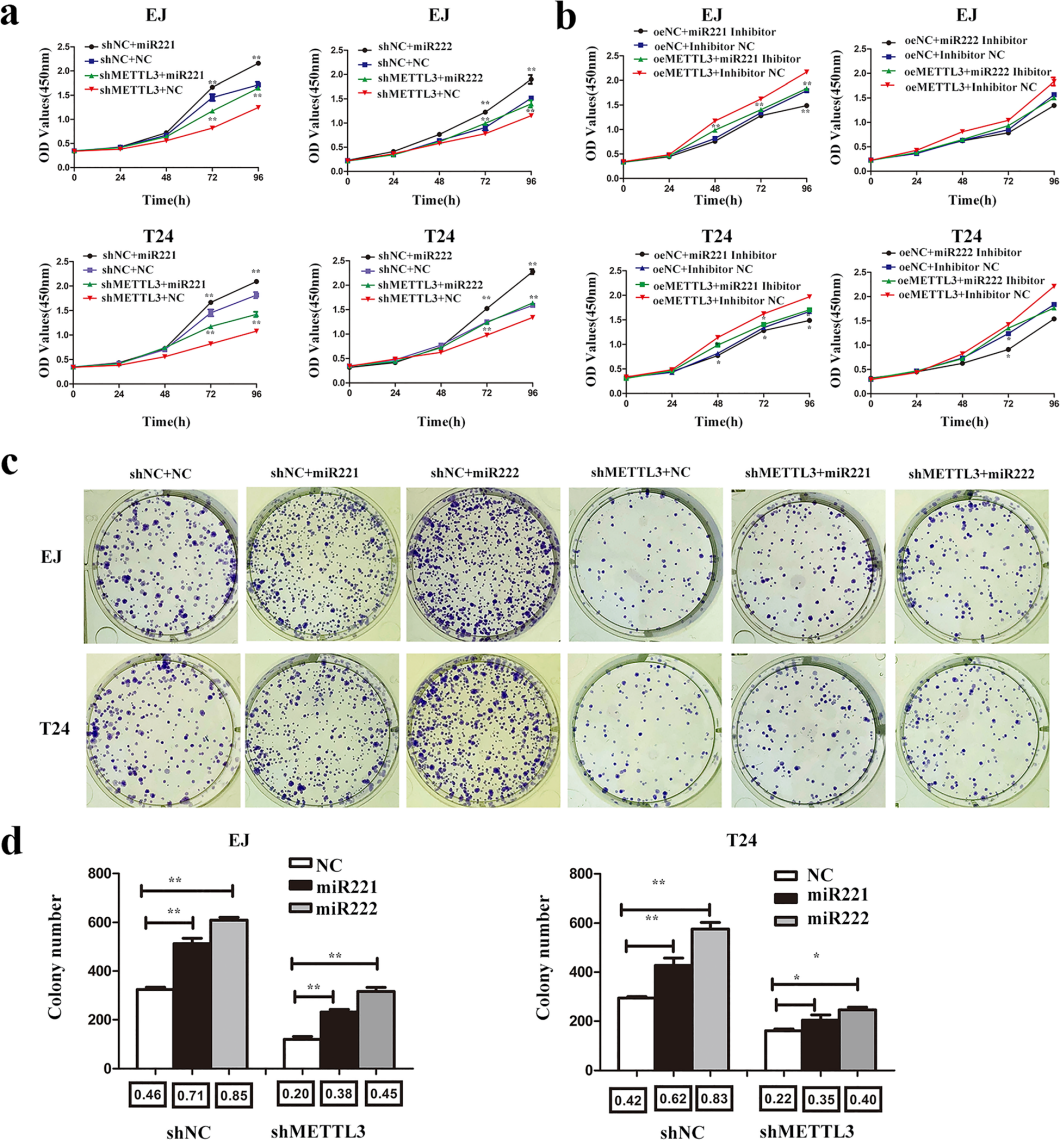
miR221 and miR222 rescued the proliferating function induced by METTL3 in bladder cancer cells. **a** and **b**. CCK8 assays were used to measure the effect of miR221/222 mimics or inhibitors on EJ and T24 cells with METTL3 knockdown and overexpression. miR221/222 mimics could **rescue** the cell growth inhibited by knockdown of METTL3 in EJ and T24 cells (**a**). The inhibitors of miR221/222 could partly **reduced** cell growth induced by overexpression of METTL3 in EJ and T24 cells (**b**). **c** and **d**. Colony formation assays showed that miR221/222 mimics could **rescue** the cell growth inhibited by knockdown of METTL3 in EJ and T24 cells. Data represented the mean ± SD from three independent experiments, *P < 0.05, **P < 0.01

When we transfected miR221/222 mimics in METTL3 knockdown cells, we found miR221/222 mimics could partly increase the bladder cancer cells proliferation inhibited by the knockdown of METTL3 (Fig. 5a). Accordingly, miR221/222 inhibitors could partly decrease the cells proliferation induced by METTL3 overexpression (Fig. 5b). The colony formation assay also displayed that miR221/222 mimics could partly increase the bladder cancer cells colony formation efficiency inhibited by the knockdown of METTL3 (Fig. 5c, d).

### miR221/222 targeted PTEN in bladder cancer

**Fig. 6:**
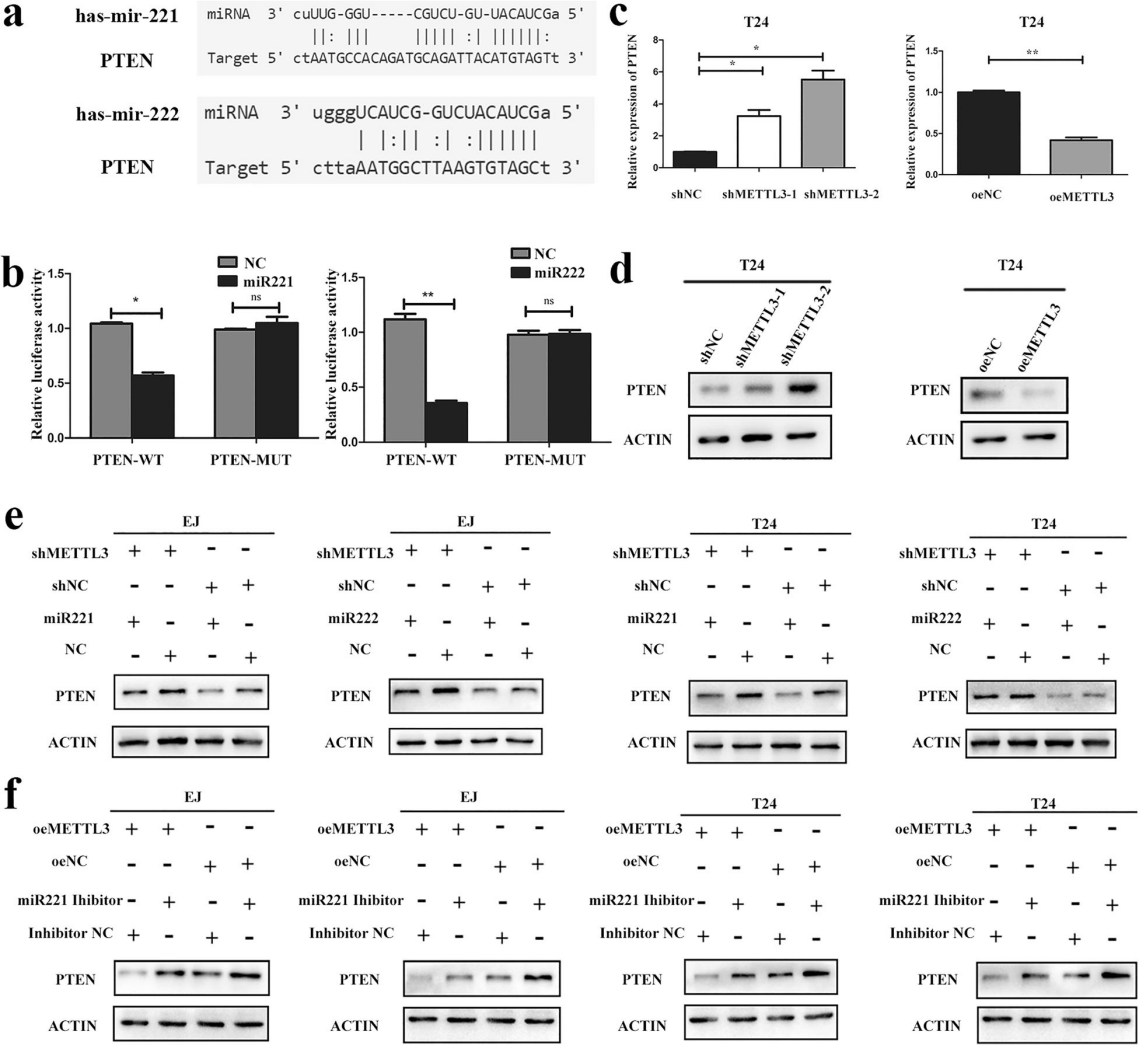
miR221 and miR222 targeted PTEN in bladder cancer. **a**. Predictive miR221/miR222 binding sites in the 3′-UTR of PTEN mRNA. **b**. Dual luciferase reporter assays demonstrated that PTEN was direct target of miR221/222 (*P < 0.05). **c** and **d**. PCR and western blot analysis showed that knockdown of METTL3 increased the PTEN expression in T24 cells, while overexpression of METTL3 in T24 cells decreased the PTEN expression. **e** and **f**. Western blot analysis showed that miR221 and miR222 mimics could partly decrease the protein expression level of PTEN induced by knockdown of METTL3 (**e**), while miR221 and miR222 inhibitors could partly increase the protein expression level of PTEN induced by overexpression of METTL3 in EJ and T24 cells (**f**)

By mirtarbase database (http://mirtarbase.mbc.nctu.edu.tw), we found that there were binding sites of miR221/222 in 3′-untranslated regions (UTR) of PTEN mRNA (Fig. 6a). In addition, dual luciferase reporter assay was performed. The wild-type 3′-UTR sequence and the mutant 3′-UTR sequence of PTEN were cloned to construct reporter plasmids and mutant vectors, respectively. We found that co-transfection of miR221/222 mimics and wild-type reporter gene plasmid decreased the luciferase activity significantly. On the contrary, there was no significant difference in luciferase activity between miR221/222 co-transfected mimics and mutant plasmids (Fig. 6b). Then we validated the expression of PTEN in METTL3 knockdown or overexpression bladder cancer cells by qRT-PCR and western blot. We found that the expression of PTEN was significantly higher in METTL3 knockdown cells, while the expression of PTEN was decreased in METTL3 overexpression cells (Fig. 6c, d). Furthermore, the expression of PTEN decreased after miR221/222 mimics transfection (Fig. 6e) and increased after miR221/222 inhibitor transfection into bladder cancer cells (Fig. 6f).

For detecting whether METTL3 may directly regulate PTEN mRNA, we mutated the binding site of the 3′-UTR region of PTEN and performed luciferase reporter assay and m6A RNA immunoprecipitation assay respectively. Results showed that there was no significant difference in luciferase activity between wild-type and mutant plasmids in bladder cancer cells (Additional file 6: Figure S4a). The MeRIP result also showed that there was no significant difference in PTEN precipitated by m6A and IgG (Additional file 6: Figure S4b). Together, these results suggested METTL3 may not directly regulate PTEN mRNA in bladder cancer.

### PTEN was negatively correlated with METTL3 expression in bladder cancer tissues

**Fig. 7:**
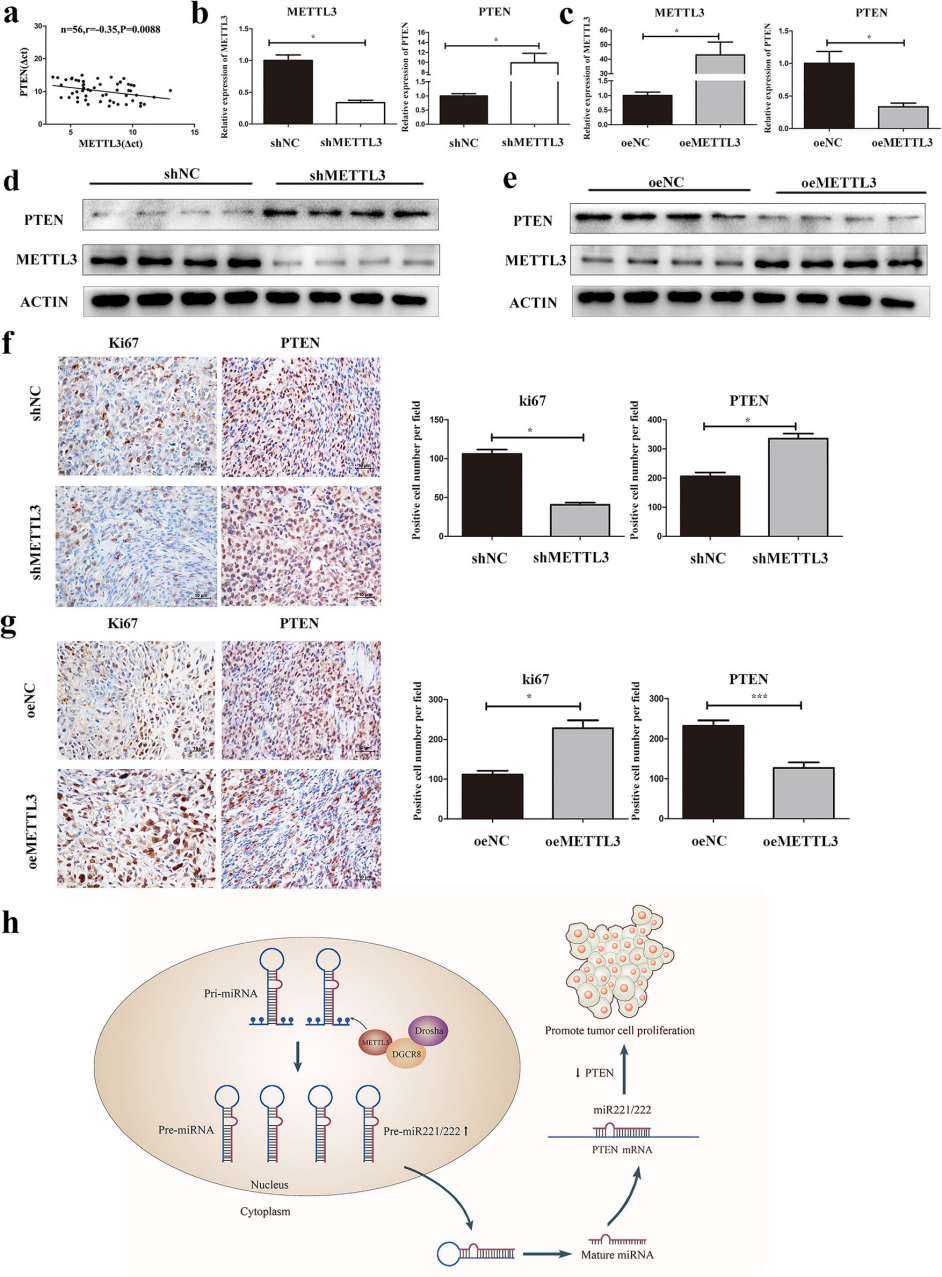
PTEN was negatively correlated with METTL3 expression in bladder cancer tissues. **a**. A moderate negative correlation between the expression of METTL3 and PTEN was showed in bladder cancer tissues by qRT-PCR. **b**, **c**, **d** and **e**. RNA and proteins was extracted from the tumors and the protein expression of METTL3/PTEN was measured using PCR and western blot. **f**. **g**. IHC analysis of ki-67, METTL3, and PTEN in xenografs (Magnification, × 400, scale bars indicated 10 μm). **h**. Mode pattern of the METTL3/miR221/222/PTEN regulatory network in bladder cancer

We investigated the expression of METTL3 and PTEN in patients’ tissues by qRT-PCR. The result showed that PTEN expression was negatively correlated with METTL3 expression in bladder cancer (Fig. 7a, r = − 0.35, *P* = 0.0088). Futhermore, we also performed qRT-PCR and western blot to detect the mRNA and protein level of METTL3 and PTEN in tissues from mouse xenograft model (Fig. 7b, c). Results showed that the mRNA level of PTEN in the tumors injected with knockdown of METTL 3(shMETTL3)significant increased than those injected with control (shNC) (Fig. 7b), while the mRNA level of PTEN in the tumors injected with METTL3 overexpression cells (oeMETTL3)significant decreased than those injected with control cells (oeNC) (Fig. 7c). In addition, the same changes were observed in PTEN protein levels in xenograft models (Fig. 7d,e). IHC of subcutaneous xenograft tumors showed that a significant decrease in the positive rate of Ki67 and a significant increase in the positive rate of PTEN in METTL3 knockdown group (Fig. 7f). The METTL3 overexpression group have the opposite result(Fig. 7g). Together, we found that PTEN was negatively correlated with METTL3 expression in bladder cancer tissues.

## Discussion

As a main component in the so-called m6A ‘writer’, METTL3 was reported to have the ability to promote bladder cancer progression via AFF4/NF-κB/MYC signaling network by an m6A dependent manner [10]. In the present study, we found METTL3 promoted bladder cancer proliferation by accelerating the maturation of pri-miR221/222 by an m6A dependent manner, resulting in the reduction of PTEN. To our knowledge, this is the first comprehensive study that METTL3 affected the tumor formation by the regulation the m6A modification in non-coding RNAs.

METTL3 was found to be upregulated in bladder cancer tissues and cell lines significantly. Cheng et al. previously found that METTL3 could promote the proliferation of bladder cancer cell lines 5637 and SV-HUC-1 [10]. In our study, we firstly verified the expression of METTL3 in six bladder cancer cell lines (EJ, T24, J82, 5637, 253 J, RT4). Then we found that METTL3 was significantly upregulated in bladder cancer cell lines, especially in EJ and T24 cell lines. As METTL3 acted as an oncogene in bladder cancer, we selected T24 and EJ cell lines with higher METTL3 expression for our follow-up experiments. Knockdown of METTL3 significantly inhibited the proliferation of T24 and EJ cell lines, whereas overexpression of METTL3 promoted the proliferation of T24 and EJ cell lines. Similarly, in vivo experiments showed that knockdown of METTL3 in T24 cells formed smaller tumors in nude mice than in the control group, while overexpression of METTL3 in T24 cells formed larger tumors in nude mice. These results confirmed the previous report that METTL3 plays an oncogenic role in bladder cancer. More importantly, we found that METTL3 expression was associated with the histological grade in bladder cancer patients. In addition, the prognosis of bladder cancer patients with high expression of METTL3 was worse, suggesting that METTL3 was a predictor of the prognosis for bladder cancer patients.

Cheng et al. found that METTL3 promoted bladder cancer progression via AFF4/NF-κB/MYC signaling network by an m6A dependent manner. In the present study, we found that METTL3 was required for the engagement of pri-miRNAs by the DGCR8 in bladder cancer. Alarcon. et al reported that METTL3 could promote the maturation of miRNAs, including let-7e, miR221/222, miR4485, miR25, miR93, miR126 and miR335 [24]. Then, we validated the above-mentioned miRNAs in T24 cell and we found that miRNAs (let-7e, miR221/222, miR4485, miR25, miR93) were significantly altered by METTL3. However, miR126 and miR335 did not change significantly, possibly due to their low levels in bladder cancer cell. Then, we predicted the prognosis of these miRNAs in bladder cancer in the database (http://kmplot.com/analysis/) and found that only miR221/222 played an oncogenic role in bladder cancer. Then, we verified the expression of miR221/222 and pri-miR221/222 in the METTL3 knockdown and overexpression bladder cancer cells. As expected, knockdown METLL3 increased the expression of pri-miR221/222, while decreased the expression of miR221/222, significantly. While, overexpression METTL3 gave the opposite result. We also found that METTL3 and miR221/222 were positively correlated in bladder cancer patients’ tissues. Moreover, METTL3 could interact with the microprocessor protein DGCR8 and positively modulating the pri-miR221/222 process in an m6A-dependent manner by further immunoprecipitated and MeRIP assay. When we transfected miR221/222 mimics or inhibitors into METTL3 knockdown and overexpression bladder cancer cells, respectively, we found that miR221/222 interference could rescue the proliferation induced by METTL3 in bladder cancer cells. All the results implied that METTL3 could promote the maturation of pri-miR221/222 in bladder cancer by m6A dependent manner.

miRNAs are small non-coding RNAs that perform biological functions by inducing degradation of mRNA targets or blocking translation to inhibit gene expression [31]. Because of the structural and functional similarities between miR221 and miR222, they are often studied together as miR221/222. miR221/222 has been reported to play an important role in the occurrence and development of many cancers such as bladder cancer [32], gastric cancer [33], breast cancer [34], thyroid cancer [35], prostate cancer [36], and so on [37, 38]. MiR221/222 are crucial molecules targeting anti-oncogenes (including p27, p57 and PTEN) in multiple cancers [32–38]. Garofalo M et al. reported that 3’UTRs of human PTEN (nucleotides 200–207; NM_000314) and human TIMP3 (nucleotides 2443–2449; NM_000362) contained regions that matched the seed sequences of miR-221 and miR-222 by bioinformatics search (Targetscan, Pictar, RNhybrid) [39]. PTEN was validated to be key molecules in regulating bladder cancer. The loss of PTEN is associated with adverse prognosis, which is expected to be crucial therapeutic target for bladder cancer [40, 41]. Additionally, early studies demonstrated that multiple oncogenic miRNAs promoted malignancy in tumors by neutralizing PTEN expression [42]. The combination of miR221/222 and PTEN has also been reported in liver cancer [39], lung cancer [39], gastric cancer [43] and thyroid cancer [35]. In the present study, the luciferase reporter assay confirmed that miR221/222 could bind to 3′-UTR of PTEN mRNA in bladder cancer. Further functional studies also verified that miR221/222 promoted tumor progression by directly targeting PTEN in bladder cancer. Therefore, we presented PTEN as a targeted gene by miR221/miR222 in bladder cancer.

Vu LP. et al have reported that METTL3 can directly regulate PTEN mRNA in the human acute myeloid leukemia MOLM-13 cell line [25]. In my study, we mutated the binding site of the 3′-UTR region of PTEN for luciferase reporter assay and m6A RNA immunoprecipitation. Results suggested that METTL3 may not directly regulate PTEN mRNA in bladder cancer. Furthermore, we found that METTL3 and PTEN were negative correlated in bladder cancer patients’ tissues. In bladder cancer cells, we also found that the expression of PTEN was significantly higher in METTL3 knockdown cells, while the expression of PTEN was decreased in METTL3 overexpression cells by qRT-PCR and western blot. Therefore, METTL3 promote the maturation of miR221/222 which could target PTEN, leading to the decrease of PTEN mRNA expression, and ultimately the decrease of PTEN protein expression.

In summary, our results showed that METTL3 was significantly increased in bladder cancer and correlated with poor prognosis of bladder cancer patients. Moreover, METTL3 could promote cell proliferation both in vitro and tumorigenesis in vivo. And we demonstrated that METTL3 could downregulate PTEN expression by interacting with the microprocessor protein DGCR8 and positively modulating the pri-miR221/222 process in an m6A-dependent manner, which may provide a prognostic and/or therapeutically target for the treatment of bladder cancer.

## Additional files

Additional file 1: **Table S1.** Clinical pathological parameters and prognostic data of bladder cancer patients. (XLS 58 kb)

Additional file 2: **Table S2.** Oligonucleotide sequences used in this study (DOCX 20 kb)

Additional file 3: **Figure S1.** Database analysis of METTL3 in bladder cancer (TIF 2008 kb)

Additional file 4: **Figure S2.** The efficiency of METTL3 knockdown and overexpression in bladder cancer cell lines. (TIF 5257 kb)

Additional file 5: **Figure S3.** Kaplan-Meier survival curves of overall survival of miRNAs and the mRNA expression of miRNAs in bladder cancer (TIF 590 kb)

Additional file 6: **Figure S4.** The relationship between METTL3 and PTEN (TIF 1597 kb)

## Data Availability

All data generated or analyzed during this study are included either in this article or in the supplementary information files.

## Abbreviations

ceRNA: competing endogenous RNA
DGCR8: The DGCR8 microprocessor complex subunit (DiGeorge syndrome chromosomal [or critical] region 8) is a protein that in humans is encoded by the DGCR8 gene
EJ, T24, J82, 5637, 253J, RT4 SV-HUC-1: human bladder cancer cell
MeRIP: m6A RNA immunoprecipitation assay
METTL3: Methyltransferase-like 3
miRNA: microRNA
mRNA: messenger RNA
pri-miRNA: primary microRNA
PTEN: phosphatase and tensin homologue
