# Supplementary material for: METTL3 promote tumor proliferation of bladder cancer by accelerating pri-miR221/222 maturation in m6A-dependent manner
Source: Mol Cancer. 2019 Jun 22;18:110. doi: 10.1186/s12943-019-1036-9 (PMC6588935; doi:10.1186/s12943-019-1036-9)
Supplement: Supplementary file 2 — Table S2. Oligonucleotide sequences used in this study (DOCX 20 kb) [file 12943_2019_1036_MOESM2_ESM.docx]

**Supplementary Table S2** Oligonucleotide sequences used in this study

| Primes and probes |  | |  | | Sequences |
| --- | --- | --- | --- | --- | --- |
| Hsa-miR-221(mimics) | | Forward | | 5′-AGCUACAUUGUCUGCUGGGUUUC-3′ | |
|  |  | Reverse | | 5′-AACCCAGCAGACAAUGUAGCUUU-3′ | |
| Hsa-miR-222(mimics) | | Forward | | 5′-AGCUACAUCUGGCUACUGGGU-3′ | |
|  |  | Reverse | | 5′-CCAGUAGCCAGAUGUAGCUUU-3′ | |
| NC(mimics) | | Forward | | 5′-UUCUCCGAACGUGUCACGUTT-3′ | |
|  |  | Reverse | | 5′-ACUUGACACGUUCGGAGAATT-3′ | |
| Hsa-miR-221(inhibitor) | |  | | 5′-GAAACCCAGCAGACAAUGUAGCU-3′ | |
| Hsa-miR-222(inhibitor) | |  | | 5′-ACCCAGUAGCCAGAUGUAGCU-3′ | |
| Inhibitor NC | |  | | 5′-CAGUACUUUUGUGUAGUACAA-3′ | |
| METTL3 | | Forward | | 5′-AAGCTGCACTTCAGACGAAT -3′ | |
|  |  | Reverse | | 5′-GGAATCACCTCCGACACTC -3′ | |
| β-actin | | Forward | | 5′-ACTGGAACGGTGAAGGTGAC-3′ | |
|  |  | Reverse | | 5′-AGAGAAGTGGGGTGGCTTTT-3′ | |
| PTEN | | Forward: | | 5′-TGGCGGAACTTGCAATCCTCAGT-3′ | |
|  |  | Reverse | | 5′-TCCCGTCGTGTGGGTCCTGA-3′ | |
| miR221 | | Forward | | 5′-GAAACCCAGCAGA-3′ | |
|  |  | Reverse | | 5′-CAATGTAGCT-3′ | |
| miR222 | | Forward | | 5′-GAGACCCAG-3′ | |
|  |  | Reverse | | 5′-TAGCCAGATGTAGCT-3′ | |
| U6 | | Forward | | 5′-CGAGCACAGAATCGCTTCA-3′ | |
|  |  | Reverse | | 5′-CTCGCTTCGGCAGCACATAT-3′ | |
