# Supplementary figures and images for: METTL3 promote tumor proliferation of bladder cancer by accelerating pri-miR221/222 maturation in m6A-dependent manner
Source: Mol Cancer. 2019 Jun 22;18:110. doi: 10.1186/s12943-019-1036-9 (PMC6588935; doi:10.1186/s12943-019-1036-9)

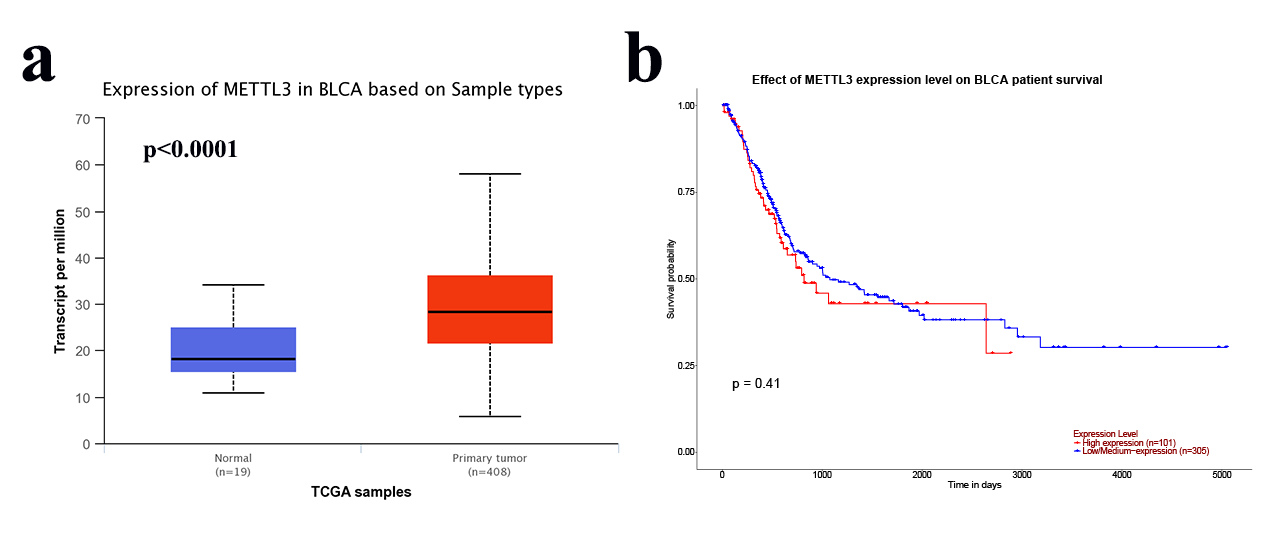

Supplement: Supplementary file 3 — Figure S1. Database analysis of METTL3 in bladder cancer (TIF 2008 kb) [file 12943_2019_1036_MOESM3_ESM.tif]

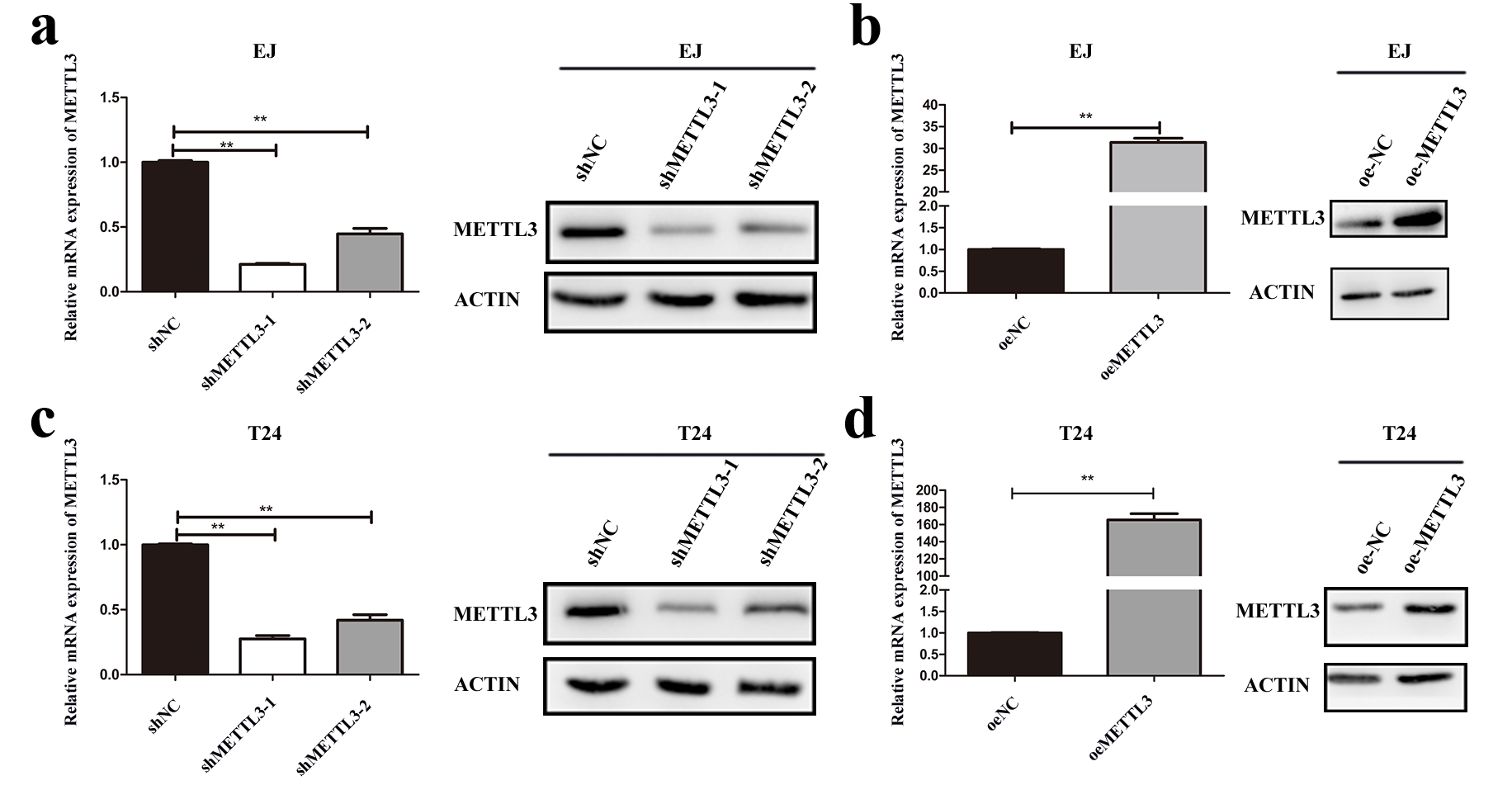

Supplement: Supplementary file 4 — Figure S2. The efficiency of METTL3 knockdown and overexpression in bladder cancer cell lines. (TIF 5257 kb) [file 12943_2019_1036_MOESM4_ESM.tif]

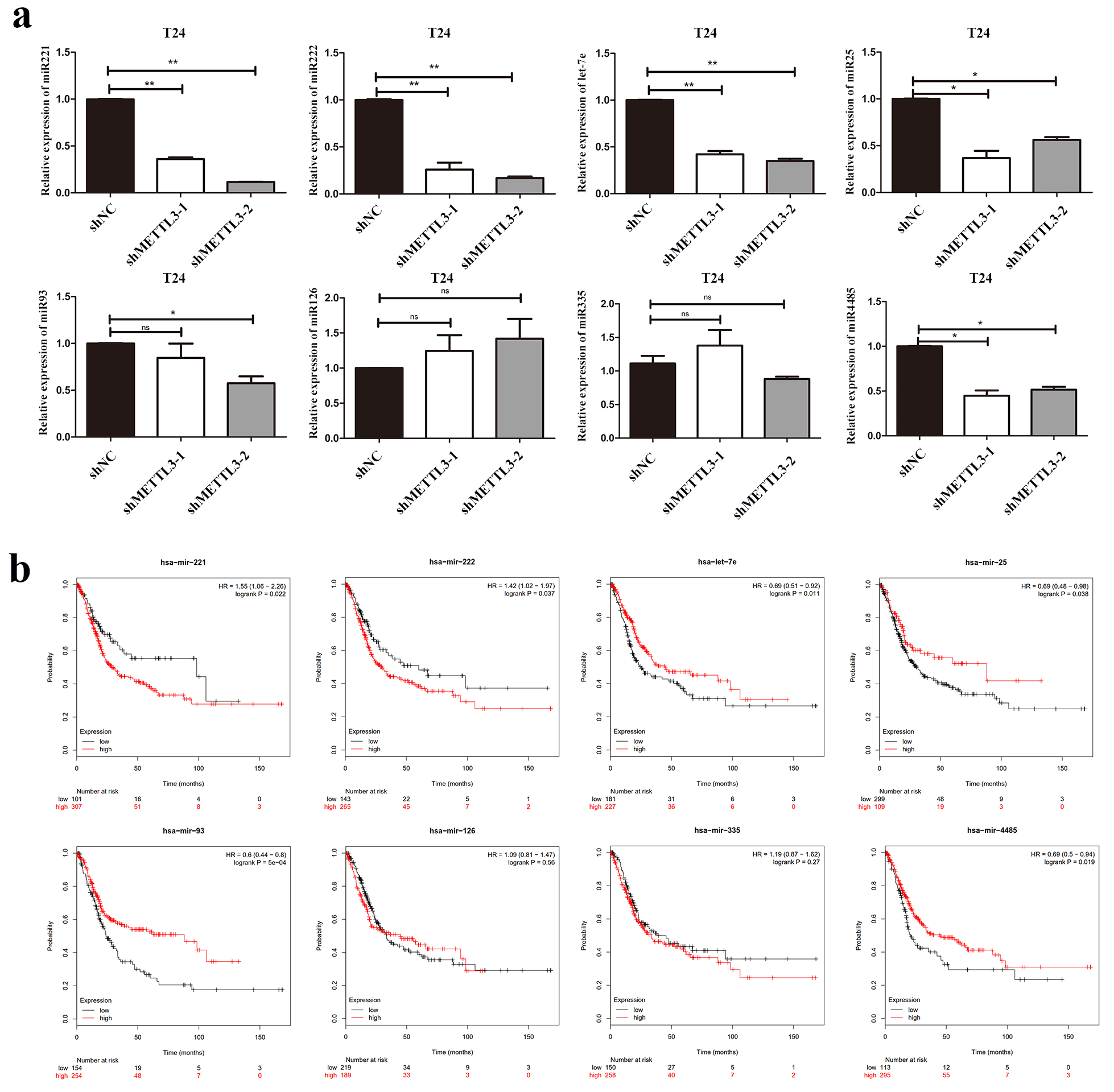

Supplement: Supplementary file 5 — Figure S3. Kaplan-Meier survival curves of overall survival of miRNAs and the mRNA expression of miRNAs in bladder cancer (TIF 590 kb) [file 12943_2019_1036_MOESM5_ESM.tif]

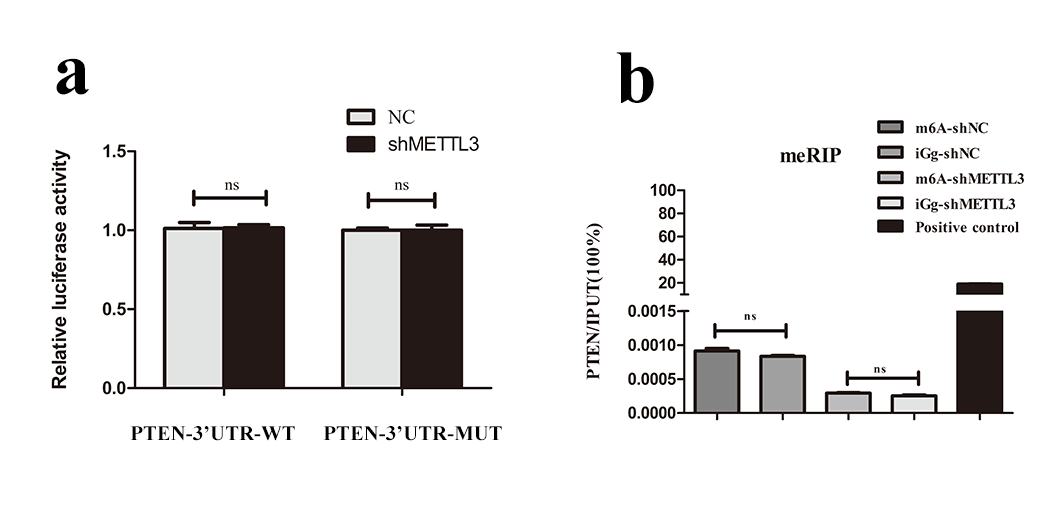

Supplement: Supplementary file 6 — Figure S4. The relationship between METTL3 and PTEN (TIF 1597 kb) [file 12943_2019_1036_MOESM6_ESM.tif]
